# Job characteristics of a Malaysian bank’s anti-money laundering system and its employees’ job satisfaction

**DOI:** 10.12688/f1000research.73234.2

**Published:** 2022-09-12

**Authors:** Rathimala Kannan, Yonesh Reddiar, Kannan Ramakrishnan, Marrynal S Eastaff, Shobana Ramesh

**Affiliations:** 1 Department of Information Technology, Faculty of Management, Multimedia University, Cyberjaya, Selangor, 63100, Malaysia; 2 Anti-Money Laundering Specialist, Standard Chartered Global Business Services, Kuala Lumpur, Kuala Lumpur, 57000, Malaysia; 3 Faculty of Computing and Informatics, Multimedia University, Cyberjaya, Selangor, 63100, Malaysia; 4 Department of Information Technology, Hindusthan College of Arts and Science, Coimbatore, Tamil Nadu, 641028, India; 5 PG and Research Department of Management Studies, Hindusthan College of Arts and Science, Coimbatore, Tamil Nadu, 641028, India

**Keywords:** Anti-money laundering system, employees’ satisfaction, job characteristic model, job diagnostic survey, Malaysia

## Abstract

**Background: **Banks and financial institutions are vulnerable to money laundering (ML) as a result of crime proceeds infiltrating banks in the form of significant cash deposits. Improved financial crime compliance processes and systems enable anti-ML (AML) analysts to devote considerable time and effort to case investigation and process quality work, thereby lowering financial risks by reporting suspicious activity in a timely and effective manner. This study uses Job Characteristics Theory (JCT) to evaluate the AML system through the job satisfaction and motivation of its users. The purpose of this study is to determine how satisfied AML personnel are with their jobs and how motivated they are to work with the system.

**Methods:** This cross-sectional study used JCT to investigate the important elements impacting employee satisfaction with the AML system. The five core dimensions of the job characteristics were measured using a job diagnostic survey. The respondents were employees working in the AML department of a Malaysian bank, and the sample group was chosen using a purposive sampling approach. A total of 100 acceptable replies were gathered and analysed using various statistical approaches. A motivating potential score was generated for each employee based on five main job characteristics.

**Results:** Findings revealed that five core job characteristics, namely, skill diversity, task identity, task importance, autonomy and feedback, positively influence the AML system employees’ job satisfaction. However, skill variety and autonomy are found to be low, which are reflected in the poor motivating potential score.

**Conclusion:** This study examined the characteristics of the AML system and its users’ job satisfaction. Findings revealed that task significance is the most widely recognised characteristic, followed by feedback and task identity. However, there is a lack of skill variety and autonomy, which must be addressed to improve employee satisfaction with the AML system.

## Introduction

By 2026, the worldwide anti-money laundering (AML) market is expected to reach $3.7 billion, with a compound annual growth rate (CAGR) of 18.3% throughout the projected period ( Kbvresearch, 2020). Money laundering (ML) is a global concern to financial institutions, particularly the banking industry, because it can weaken institutions and subject them to major risks, including operational, legal and reputational risks ( Mohamad Abdul Latif and Abdul-Rahman, 2018; Raweh, Erbao, and Shihadeh, 2017). Combating crime is one of the Sustainable Development Goals (SDGs). The United Nations has developed a series of objectives aimed at reducing criminal behaviour globally as part of Goal 16. Crime reduction is a vital step in laying the way for long-term development because this action helps establish stable communities, enhance effective government and improve people’s wellbeing ( Canhoto, 2021).

ML is the process of converting illegally obtained money into legitimate funds that appear to have come from legal sources ( Zaman *et al.*, 2020). Accordingly, understanding how money is laundered is crucial to comprehending economic growth ( Alnasser Mohammed, 2020). Banks and financial institutions help people move money globally and keep track of their clients’ identities and financial habits. Consequently, governments throughout the world have recruited their help in detecting and preventing ML, which is a critical instrument in long-term economic development and the battle against crime ( Zaman *et al.*, 2020). The increasing amount of global transactions has prompted the implementation of anti-ML (AML) solutions in banks and financial institutions.

### Anti-money laundering system (AMLS)

AML is a comprehensive term that refers to methods, processes, rules and regulations aimed at preventing the illicit or criminal generation of funds ( Choo *et al.*, 2014). All governments, including Malaysia, have made the battle against ML and terrorist financing a top priority. Since 2001, the Malaysian government and law enforcement agencies have been attempting to confront ML. An AML system (AMLS) enables banks and other financial institutions to use automated procedures to monitor client behaviour for alleged illicit financial activity. Although such an effort is generally beneficial, their success should be evaluated before strengthening AML regulation.

### Employees’ job satisfaction and motivation

Job satisfaction is a psychological notion generally determined by employees’ subjective feelings. Job satisfaction is influenced by a variety of independent variables. Educational credentials, nature of work, remuneration, job stability, promotion chances and family and work-life balance are factors to consider ( Nadaf, 2018). On a personal level, job satisfaction considers the type of employment, recognition and freedom, as well as a wide range of duties, social position, moral ideals, social values, authority, ability, responsibility, creativity and accomplishment ( Gudayehu gomeshu and Adisu Fanta, 2018). The existing literature has shown that JCT is applied to study job satisfaction of enterprise resource planning system users ( Liere-Netheler *et al.*, 2017). These studies have found technology characteristics explaining job satisfaction of the users. Another study has investigated the impact of job characteristics on employee satisfaction at public radio stations, and discovered that five core characteristics have a strong positive link with job satisfaction ( Khalil, 2017). Job satisfaction amongst bank employees is important because certain job characteristics are highly appealing and contribute to satisfaction, as well as aspects of jobs that cause dissatisfaction. To ensure the efficiency and productivity of employees, factors contributing to job satisfaction and those that can lead to job dissatisfaction should be understood ( Nadaf, 2018).

Job characteristics theory (JCT) of Hackman and Oldham is extensively used as a paradigm for studying how certain employment characteristics impact job outcomes, such as job satisfaction ( Tang and Do, 2019). JCT offers suggestions for improving job enrichment in organisations. According to this theory, five factors—skill variety, task identity, task significance, autonomy, and feedback—have an impact on motivation, satisfaction with work, and performance. This paradigm has been used to discover the best method for designing work so that employees are internally motivated and perform job effectively. JCT can be used to examine how the current AMLS (anti-money laundering system) is designed and what can be improved in order to increase employee’s satisfaction solely from the job characteristics perspective ( Tang and Do, 2019). This is appropriate because the AMLS is primarily used by bank employees to detect money laundering transactions.

Malaysia has implemented a comprehensive AML/Combating the Financing of Terrorism (AML/CFT) framework, although its efficiency is debatable owing to the low number of ML prosecutions ( Syed Mustapha Nazri, Zolkaflil, and Omar, 2019). Malaysia lacks an effective investigative support structure to assist law enforcement agencies in their investigations ( Zolkaflil, Omar, and Syed Mustapha Nazri, 2019). The case investigation process is one of the primary issues noted in the AML Department. The investigation process takes longer time because of the necessity to access different internal source systems. In terms of case resolution time, this drawback results in low productivity and ageing. Case investigation quality has likewise deteriorated, resulting in a high monthly error rate score. Process gaps and superfluous steps are present, resulting in poor work process and flow. However, limited research has indicated that obtaining information is one of the obstacles that law enforcement organisations confront whilst investigating ML ( Zolkaflil *et al.*, 2019).

The purpose of this study is to use JCT to examine the job satisfaction of AML department employees. The respondents’ respective motivating potential score is calculated to measure their motivational levels in executing their job with AMLS. This research is a quantitative and cross-sectional study. A job diagnostic survey was adopted as the survey instrument, and 100 valid responses were obtained for further analysis. This research contributes to an improved understanding of AMLS’ performance and the satisfaction and motivation of its employees in detecting ML activities.

## Methods

### Research methods

This study used a positivism research paradigm, which breaks down complex world scenario into variables. The current research is a quantitative and cross-sectional study ( Obodo, Okonkwo, and Aboh, 2019). The important aspects determining employee satisfaction with AMLS were investigated using JCT. The five key job elements of the job characteristics model were assessed using a job diagnostic survey ( Liere-Netheler *et al.,* 2017; Moras and Kashyap, 2021). Accordingly, the current study used and modified a job diagnostics survey instrument ( Sinha and Bhatt, 2020). The questionnaire had two parts: Part A consists of questions related to demographic data, and Part B comprises 23 questions adopted and adapted from the job diagnostic survey. Each category’s items were graded using a five-point Likert scale ( Khalil 2017; Obodo *et al.*, 2019): 1 = strong disagreement, 2 = disagreement, 3 =, 4 = agreement and 5 = strong agreement. The respondents were employees who work in the AML department of one bank, and the sample population was selected using a purposive sampling method ( Moras and Kashyap, 2021). The questionnaire was created in Google forms and distributed through online to the employees of AML department of the bank from 1 ^st^ November 2018 until 31 ^st^ December 2018. 400 employees were working in the department during the data collection period and the questionnaire was sent out to all of them. Table 1 presents Job diagnostic survey questions used in this study. Yonesh Reddiyar, a co-author of this paper, was working in the AML department of the bank and he distributed the questionnaire online and sent reminders to his colleagues at the end of two months. A total of 100 valid responses were collected. Based on JCT, the score for five job characteristics; skill variety, task identity, task significance, autonomy and feedback are calculated. Also, a motivating potential score (MPS) was calculated for each employee based on these five characteristics and further explained in the results section.

**Table 1.** Job diagnostic survey questions used in this study.

No	Questions
1	I have almost complete responsibility for deciding how and when the work is to be done.
2	I have a chance to do a number of different tasks, using a wide variety of different skills and talents.
3	I do a complete task from start to finish. The results of my efforts are clearly visible and identifiable.
4	What I do affects the well-being of other people in very important ways.
5	My manager provides me with constant feedback about how I am doing.
6	The work itself provides me with information about how well I am doing.
7	I make insignificant contributions to the final product or service.
8	I get to use a number of complex skills on this job.
9	I have very little freedom in deciding how the work is to be done.
10	Just doing the work provides me with opportunities to figure out how well I am doing.
11	The job is quite simple and repetitive.
12	My supervisors or coworkers rarely give me feedback on how well I am doing the job.
13	What I do is of little consequence to anyone else.
14	My job involves doing a number of different tasks.
15	Supervisors let us know how well they think we are doing.
16	My job is arranged so that I do not have a chance to do an entire piece of work from beginning to end.
17	My job does not a l low me an opportunity to use discretion or participate in decision making.
18	The demands of my job are highly routine and predictable.
19	My job provides few clues about whether I'm performing adequately.
20	My job is not very important to the company's survival.
21	My job gives me considerable freedom in doing the work.
22	I have almost complete responsibility for deciding how and when the work is to be done.
23	I have a chance to do a number of different tasks, using a wide variety of different skills and talents.

**Ethics approval and consent**

Ethical standards were respected throughout the research process; the Technology Transfer Office, secretariat of research ethics committee, Multimedia University, approved the study protocol and informed consent procedures with file No. EA1332021.

### Research framework

JCT was developed by Hackman and Oldham in 1976 and modified in 1980 to provide ideas on job specifications that will meet the needs of organisations and the people doing the job ( Tang and Do, 2019). This theory has been used as a management framework to determine how specific job features influence employment results. JCT describes the relationship between job features and individual responses to work or tasks at hand. Moreover, JCT posits skill variety, task identity, task significance, autonomy and feedback as the five core job dimensions that influence outcomes of motivation, satisfaction and performance.

Skill variety refers to the extent to which a work demands a variety of diverse actions to be done, necessitating the use of different abilities and talents from individuals. Hence, the first hypothesis is formulated as follows:

H1: Skill variety in AMLS positively influences employees’ job satisfaction.

Task identity is referred to as the degree to which a job necessitates the execution of a complete and identifiable piece of work (i.e. executing a job from start to finish with a visible end). Thus, the second hypothesis is formulated as follows:

H2: Task identity in AMLS positively influences employees’ job satisfaction.

Task significance is the degree to which a job has a significant influence on other people’s lives or work, whether in the immediate organisation or external world. Following these characteristics, the third hypothesis is formulated as follows:

H3: Task significance in AMLS positively influences employees’ job satisfaction.

Autonomy is referred to as the degree to which jobs give people considerable flexibility, independence, and judgement in arranging the work and deciding the processes to be utilised in carrying it out. Accordingly, the fourth hypothesis is derived as follows:

H4: Autonomy in AMLS positively influences employees’ job satisfaction.

Feedback is the degree to which employees receive clear information on their performance. Thus, the fifth hypothesis is formulated as follows:

H5: Feedback in AMLS positively influences employees’ job satisfaction.

**Figure 1.**
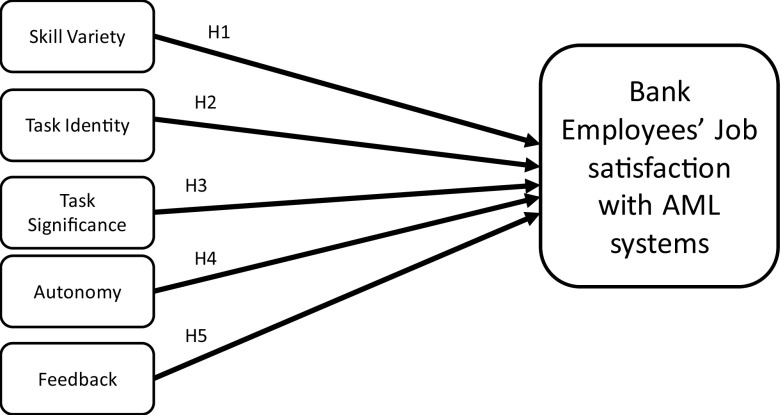
Research methods.

Figure 1 depicts the theoretical research framework and hypotheses formulated based on JCT.

## Results

A total of 100 legitimate replies were collected from bank employees in a Malaysian bank’s anti-ML department. No questionnaire responses were excluded for further analysis. Descriptive statistics shows that 55% male and 45% female employees participated in the survey. The majority are between 22 to 29 years old, and 36% of them have under 2 years of experience with AML department, while 51% have 2 to 5 years of experience. Moreover, the majority of these employees are specialists (54%) and senior analysts (42%). Table 2 illustrates the descriptive statistics of the respondents. Table 3 explains how the five core job characteristics scores are calculated.

**Table 2.** Descriptive statistics of the AML department employees’ demography.

Variable	Options	Percentage
Gender	Male	55
	Female	45
Age	22-29 years	82
	30-39 years	17
	40-49 years	1
Year of employment	Less than 2 years	36
	2-5 years	51
	More than 5 years up to 10 years	11
	More than 10 years	2
Job position	Junior Analyst	1
	Senior Analyst	42
	Specialist	54
	Other	3

**Table 3.** Calculation of job characteristic scores for 100 respondents.

Job characteristics	Formula to calculate the score for each respondents	Average score	Standard deviation	Variance	Skewness
Skill variety	(Q2 + Q8 + (6 – Q11) + Q14 + (6 – Q18)) /5	2.058	0.122	0.015	2.235
Task identity	(Q3 + (6 – Q7) + (6 – Q16) + Q22) /4	3.5275	0.117	0.014	2.763
Task significance	(Q4 + (6 – Q13) + (6 – Q20) + Q23) /4	3.95	0.142	0.02	−3.029
Autonomy	(Q1 + (6 – Q9) + (6 – Q17) + Q21) /4	2.06	0.163	0.027	3.04
Feedback	(Q5 + Q6 + Q10 + (6 – Q12) + Q15 + (6 – Q19)) /6	3.565	0.157	0.025	−1.526

**Figure 2.**
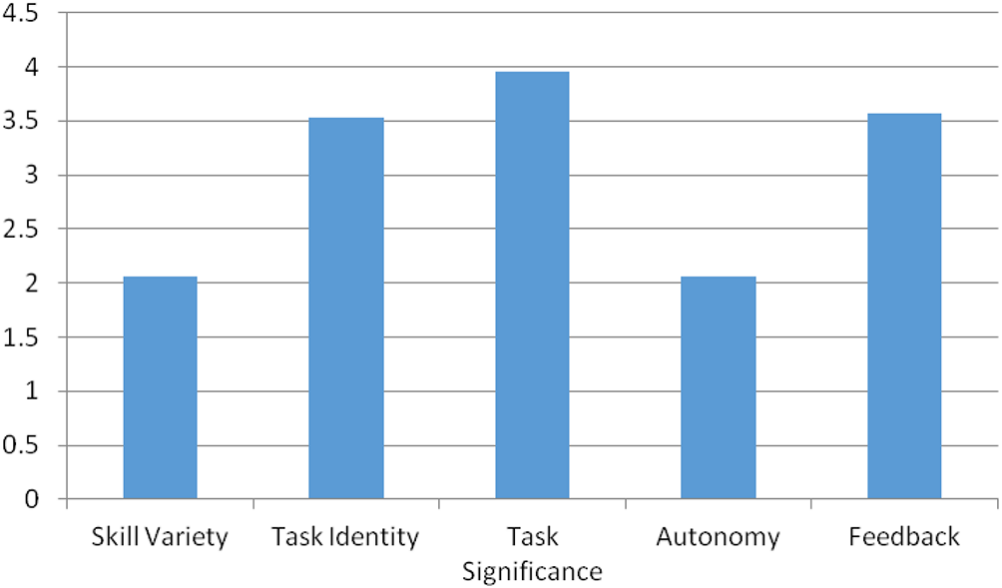
Five core job characteristics of the AML system.

Table 4 shows the frequency distribution of the five core variables, which indicate how respondents feel about the AML system and how satisfied they are with their jobs. The importance of the task significance is clearly understood by the majority of respondents, with 87 percent of employees giving it a score of 4. However, 78 percent of employees gave a score of 2 to skill variety, and 85 percent of employees gave a score of 2 to autonomy. Figure 2 compares all five job characteristics variables and clearly shows that the AML system lacks skill variety and autonomy.

**Table 4.** Job characteristics score distribution.

Job characteristics	Frequency distribution	
	score	%
Skill variety	2.0	78
	2.2	16
	2.4	5
	2.6	1
Task identity	3.5	89
	3.75	5
	4.0	4
	3.25	2
Task significance	4.0	87
	3.75	7
	3.5	5
	3.25	1
Autonomy	2.0	85
	2.25	9
	2.75	3
	2.5	3
Feedback	3.667	63
	3.5	20
	3.333	11
	3.167	5
	3	1

Table 5 shows that the five hypotheses are validated, indicating that the five core work qualities have a favourable impact on the job satisfaction of AML department personnel. This finding is consistent with a previous study ( Khalil, 2017). The JCT states that the overall ability of a job to internally motivate employees is high when the following conditions are met; Firstly, among skill diversity, task significance, and task identity one or more should be high. Secondly the job autonomy is high. Thirdly the job is high on feedback. The extent to which the aforementioned requirements are accomplished is gauged by the motivational potential score (MPS) which is calculated as shown below.

$$\mathrm{MPS}=\frac{\text{Skill Variety}+\text{Task Significance}+\text{Task Identity}}{3}*\text{Autonomy}*\text{Feedback}$$

**Table 5.** Results of hypothesis testing.

Hypothesis	Unstandardized estimates	Standard errors	Critical values	P-value	Results
H1	2.237102	0.086456	25.875745	0.000	Supported
H2	2.383759	0.086918	27.425406	0.000	Supported
H3	2.389284	0.070730	33.780395	0.000	Supported
H4	11.531509	0.064338	179.233030	0.000	Supported
H5	6.585809	0.064003	102.897783	0.000	Supported

**Figure 3.**
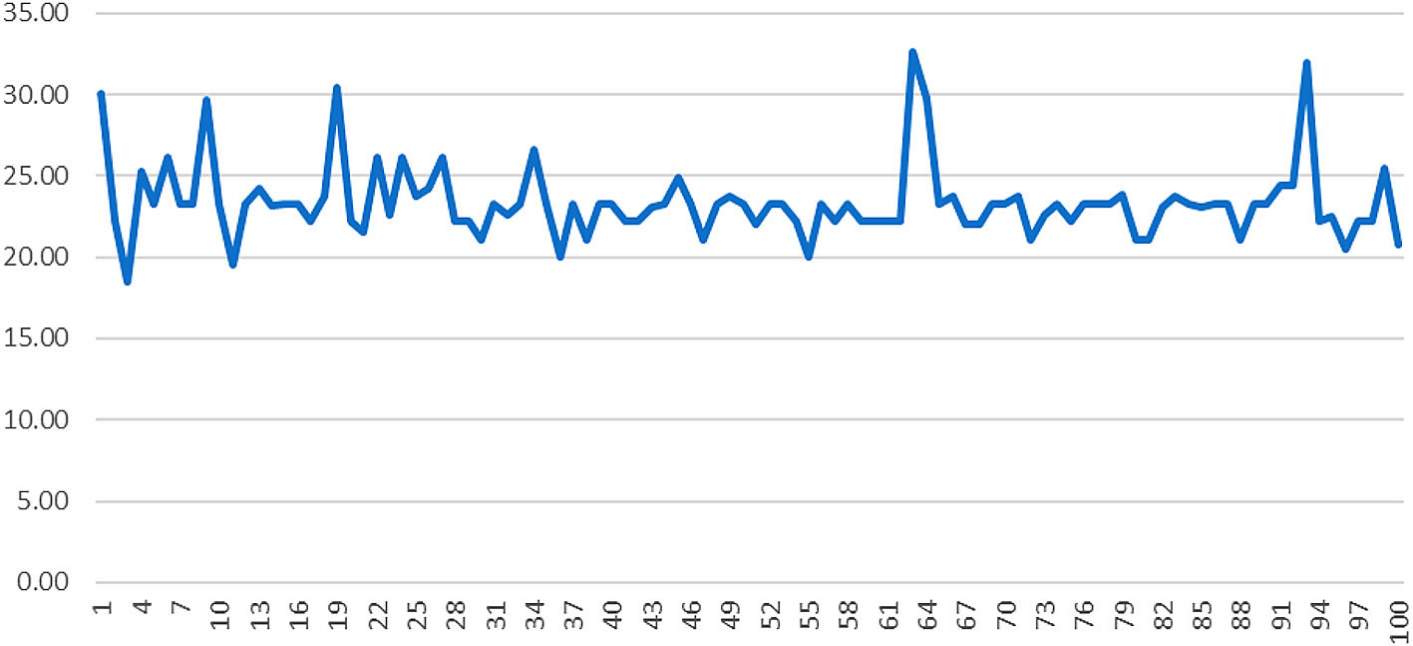
Motivating potential score of AML employees.

Given that the MPS formula is multiplicative, employees’ MPS score would be 0 if they answered 0 for autonomy, feedback or the sum of skill variety, task identity and task significance ( Bahrami *et al.*, 2016). Figure 3 shows the motivating potential score of AML employees: the majority of them had MPS values of 20 to 30 out of 125, which is considered low.

## Discussion and implications

This study aims to assess the job satisfaction and motivational index of AML department employees. JCT maps the five core job dimensions into three psychological states, resulting in or influencing five work-related consequences ( Sinha and Bhatt, 2020). Skill variety, task identity and task significance are mapped to experienced meaningfulness of work. Autonomy is mapped to experienced responsibility of the outcomes of work ( Obodo *et al.*, 2019). Feedback from the AML department is mapped to knowledge of the actual results of work activities.

The outcome of this study has the following implications.

1. Skill Variety: This section demonstrates that the AML department job does not necessitate diverse actions in carrying out the task, requiring the use of a variety of skills and abilities. Generally, the AML department’s work is boring and repetitious. When employees are not given option for skill variety, their motivation is reduced ( Putra, Istiatin, and Djumali, 2020). The work is extremely straightforward, with activities carried out in a predictable and repetitive manner.
2. Task Identity: When employees in the AML department understand what has to be done and can see a partial result of what they eventually accomplished, they will feel as though they are performing an important job.
3. Task Significance: This section demonstrates that the AML work has a significant influence on the company, whether individuals are in the immediate organisation or in the larger world. The job has additional importance because it contributes to the wellbeing of those involved in the fight against ML, whether physically or psychologically. AML department employees place a high value on task significance since they are aware of how essential their job is.
4. Autonomy: This section demonstrates that personnel in the AML department do not have the flexibility to accomplish their tasks. Furthermore, employees lack the freedom to select how to carry out certain duties, as well as the extent to which they may conduct daily case investigations. This result confirms the findings of a study that used JCT to investigate job satisfaction amongst police officers ( Obodo *et al.*, 2019). These findings reveal that jobs related to the investigation of illegal activities lack autonomy, which is mapped to experience responsibility of work outcomes.
5. Feedback: This section demonstrates that personnel in the AML department have access to clear, explicit and thorough information on the efficacy of their work. They also have an improved understanding of the overall impact of their job activities and what specific measures (if any) they need to take to increase productivity.

## Conclusions

In contemporary firms, job redesign is gaining popularity as a method for seeking to both increase employee productivity and the quality of their working experience. This study aimed to examine the job satisfaction and motivation of bank AML employees from the job characteristics perspective and applied JCT-based research framework. A cross-sectional quantitative research was conducted among bank employees who work in AML department. The findings revealed that the five key job characteristics have a positive impact on AML department employees’ job satisfaction. AMLS was deemed to be deficient in skill diversity and autonomy, which are two of the five aspects. A motivating potential score is calculated which indicates to what extent AMLS characteristics internally motivates employee’s satisfaction. This research suggests that the AML department should further focus on the two job characteristics to improve employees’ job satisfaction. The current study’s shortcomings include the fact that it is a cross-sectional research using data obtained from employees of a single bank. Thus, the findings may not be applicable to other situations. This study may be replicated in a variety of settings, including alternative banking job profiles, non-banking industries and microfinance firms.

## Data availability

### Underlying data

Figshare: AML employees Job satisfaction - Raw Data. https://doi.org/10.6084/m9.figshare.15000003.v3 ( Kannan and Reddiar, 2021).

This project contains the following underlying data:

- AML employees Job satisfaction - Raw data.csv (responses from the questionnaire for the AML department employees)

Data are available under the terms of the Creative Commons Attribution 4.0 International license (CC-BY 4.0).
