# Supplementary material for: Job characteristics of a Malaysian bank’s anti-money laundering system and its employees’ job satisfaction
Source: F1000Res. 2022 Sep 12;10:1052. Originally published 2021 Oct 15. [Version 2] doi: 10.12688/f1000research.73234.2 (PMC9540008; doi:10.12688/f1000research.73234.2)
Supplement: Peer review history

# Peer review: Reviewer response for version 2

**DOI:** 10.5256/f1000research.138298.r150176

**Published:** 2022-10-06

**Authors:** Roselina Ahmad Saufi

**Affiliations:** 1 Universiti Malaysia Kelantan, Pengkalan Chepa, Malaysia; 2 Malaysian Graduate School of Entrepreneurship and Business, Kelantan, Malaysia

The authors have done the correction and editing accordingly. I am satisfied with it.

Is the work clearly and accurately presented and does it cite the current literature?

Partly

If applicable, is the statistical analysis and its interpretation appropriate?

Partly

Are all the source data underlying the results available to ensure full reproducibility?

No source data required

Is the study design appropriate and is the work technically sound?

Yes

Are the conclusions drawn adequately supported by the results?

No

Are sufficient details of methods and analysis provided to allow replication by others?

Yes

Reviewer Expertise:

Organisational Behaviour especially in Leadership and HR

I confirm that I have read this submission and believe that I have an appropriate level of expertise to confirm that it is of an acceptable scientific standard.

# Peer review: Reviewer response for version 2

**DOI:** 10.5256/f1000research.138298.r150178

**Published:** 2022-09-26

**Authors:** Henry Inegbedion

**Affiliations:** 1 Department of Business Studies, Landmark University, Ilorin, Nigeria

The authors failed to address any of the queries raised in the first review.

1. Even the simplest of the queries on:  " *1 = strong disagreement, 2 = disagreement, 3 =, 4 = agreement and 5 = strong agreement." *What is 3? They simply ignored. 
2. No attempt was made to develop the hypotheses.
3. I can’t see anything about the validity and reliability.
4. The interpretation of results should be explained too. It is not enough to indicate that the hypotheses are supported. Why are they supported? What range of p-values will make the hypotheses rejected?
5. The contribution of the study is still conspicuously missing.

Is the work clearly and accurately presented and does it cite the current literature?

Yes

If applicable, is the statistical analysis and its interpretation appropriate?

Partly

Are all the source data underlying the results available to ensure full reproducibility?

Yes

Is the study design appropriate and is the work technically sound?

Partly

Are the conclusions drawn adequately supported by the results?

Partly

Are sufficient details of methods and analysis provided to allow replication by others?

Partly

Reviewer Expertise:

Statistics, Operations Management, Marketing, Modelling and Entrepreneurship

I confirm that I have read this submission and believe that I have an appropriate level of expertise to state that I do not consider it to be of an acceptable scientific standard, for reasons outlined above.

# Peer review: Reviewer response for version 1

**DOI:** 10.5256/f1000research.76874.r147004

**Published:** 2022-09-01

**Authors:** Ehi Eric Esoimeme

**Affiliations:** 1 Cardiff Law School, University of Wales System, Cardiff, Wales, UK; 2 University of Lagos, Lagos, Nigeria; 3 E-Four and AAF, Lagos, Nigeria

This is my review:

1. The manuscript contains new and significant information to justify publication. This study proposes a hypothesis that if an anti-money laundering system (AMLS) has a proper investigation process, then bank employees would be motivated to do their job and would be satisfied. Therefore, to determine the effectiveness of AMLS, the current study uses Job Characteristics Theory (JCT) to measure bank employees’ job satisfaction and motivation levels.
2. The research problem is significantly and concisely stated in the introduction. Malaysia has implemented a comprehensive AML/Combating the Financing of Terrorism (AML/CFT) framework, although its efficiency is debatable owing to the low number of ML prosecutions. Malaysia lacks an effective investigative support structure to assist law enforcement agencies in their investigations.
3. The literature citation is adequate and sufficient. The authors used both primary and secondary data.
4. The experimental and/or theoretical methods have been described comprehensively. The current research is a quantitative and cross-sectional study using data obtained from employees of a single bank to investigate employee satisfaction with AMLS using Job Characteristics Theory (JCT).
5. Although the findings from this research will be of value to academics, policy makers and practitioners as the research contributes to an improved understanding of AMLS’ performance and the satisfaction and motivation of its employees in detecting ML activities, the focus of the research on a single bank limits its impact and effectiveness. But this can be addressed by other Researchers as the Authors have done an elaborate job with the current approach.
6. The interpretations and conclusions in this research paper are justified by the results. The findings revealed that the five key work characteristics have a favourable impact on AML department employees’ job satisfaction. AMLS was deemed to be deficient in skill diversity and autonomy, which are two of the five aspects. Consequently, this research suggests that the AML department should further focus on the two job characteristics to improve employees’ job satisfaction.
7. The language used in this paper is acceptable and the article is readable.

Is the work clearly and accurately presented and does it cite the current literature?

Yes

If applicable, is the statistical analysis and its interpretation appropriate?

Yes

Are all the source data underlying the results available to ensure full reproducibility?

Yes

Is the study design appropriate and is the work technically sound?

Yes

Are the conclusions drawn adequately supported by the results?

Yes

Are sufficient details of methods and analysis provided to allow replication by others?

Yes

Reviewer Expertise:

Money Laundering, Financial Crime, Corruption, Fraud

I confirm that I have read this submission and believe that I have an appropriate level of expertise to confirm that it is of an acceptable scientific standard.

# Peer review: Reviewer response for version 1

**DOI:** 10.5256/f1000research.76874.r147237

**Published:** 2022-08-22

**Authors:** Henry Inegbedion

**Affiliations:** 1 Department of Business Studies, Landmark University, Ilorin, Nigeria

The research problem is interesting and relevant. However, there are some shortcomings that the authors must address:

1. The research problem is not assertively articulated.
2. The study lacks the required depth of an adequate empirical review.
3. The author's methodology section is like an empirical review. In this section they are expected to indicate how they implemented the study and not what others did. Also, the hypotheses were not developed, the authors just stated them arbitrarily. The authors also failed to indicate how they conducted validity and reliability tests or why they chose not to. I missed the method of inferential tests; is it included? In defining their Likert scale items they did not indicate what 3 represents.
4. I have reservations about the interpretation of results. The authors formulated and tested alternative hypotheses and all the items in the instrument were in the affirmative form. How did the authors conclude that all the hypotheses were supported with all p values < 0.01? The interpretation needs to be revisited as it is crucial to the study's inference.
5. In the final analysis, the authors failed to indicate the contributions of the study to knowledge. What is the justification for publication? 

Is the work clearly and accurately presented and does it cite the current literature?

Yes

If applicable, is the statistical analysis and its interpretation appropriate?

Partly

Are all the source data underlying the results available to ensure full reproducibility?

Yes

Is the study design appropriate and is the work technically sound?

Partly

Are the conclusions drawn adequately supported by the results?

Partly

Are sufficient details of methods and analysis provided to allow replication by others?

Partly

Reviewer Expertise:

Statistics, Operations Management, Marketing, Modelling and Entrepreneurship

I confirm that I have read this submission and believe that I have an appropriate level of expertise to state that I do not consider it to be of an acceptable scientific standard, for reasons outlined above.

# Peer review: Reviewer response for version 1

**DOI:** 10.5256/f1000research.76874.r97580

**Published:** 2022-08-05

**Authors:** Roselina Ahmad Saufi

**Affiliations:** 1 Universiti Malaysia Kelantan, Pengkalan Chepa, Malaysia; 2 Malaysian Graduate School of Entrepreneurship and Business, Kelantan, Malaysia

**Title : Job characteristics of a Malaysian bank’s anti-money laundering system and its employees’ job satisfaction**

**Synopsis of the Study**

 The study focusses on evaluating the anti-ML (AML) system through job satisfaction and motivation of employees’ respondent working in the AML department of a Malaysian bank. This study uses Job Characteristics Theory (JCT) to evaluate the AML system through the job satisfaction and motivation of its users. The purpose of the study was to determine how satisfied AML personnel are with their jobs and how motivated they are to work with the system.

**Reviewer Comments:**

1. What is the rationalization of the five core dimensions i.e. skill variety, task identity, task significance, autonomy and feedback to have affected the ES (employee satisfaction) in the context of AMLS (anti-money laundering system) practices at the bank?
2. The authors mentioned - *"Therefore, to determine the effectiveness of AMLS, the current study uses JCT to measure bank employees’ job satisfaction and motivation levels"* – However, the study's hypotheses were focused on job satisfaction only. Please clarify and improve the statement in the article.
3. From which JDS (job diagnostic survey) did the authors modified?
4. The conclusion section to be affirmed. How do the job characteristics relate to the motivation level? and eventually relate to the job satisfaction.

I would like to request the authors to make few more affirmations on the above.

Is the work clearly and accurately presented and does it cite the current literature?

Partly

If applicable, is the statistical analysis and its interpretation appropriate?

Partly

Are all the source data underlying the results available to ensure full reproducibility?

No source data required

Is the study design appropriate and is the work technically sound?

Yes

Are the conclusions drawn adequately supported by the results?

No

Are sufficient details of methods and analysis provided to allow replication by others?

Yes

Reviewer Expertise:

Organisational Behaviour especially in Leadership and HR

I confirm that I have read this submission and believe that I have an appropriate level of expertise to confirm that it is of an acceptable scientific standard, however I have significant reservations, as outlined above.
